# Niacinamide Antimicrobial Efficacy and Its Mode of Action via Microbial Cell Cycle Arrest

**DOI:** 10.3390/microorganisms12081581

**Published:** 2024-08-02

**Authors:** Noa Ziklo, Maayan Bibi, Lior Sinai, Paul Salama

**Affiliations:** Innovation Department, Sharon Personal Care Ltd., Eli Horovitz St. 4, Rehovot 7608810, Israel; noa.ziklo@sharonpc.com (N.Z.); bibimaayan@gmail.com (M.B.); lior.sinai@mail.huji.ac.il (L.S.)

**Keywords:** niacinamide, preservation, antimicrobial efficacy, DNA interaction, DNA damage, cell-cycle arrest, cosmetic application, skincare

## Abstract

Niacinamide is a versatile compound widely used in the personal care industry for its ample skin benefits. As a precursor to nicotinamide adenine dinucleotide (NAD+), essential for ATP production and a substrate for poly-ADP-ribose polymerase-1 (PARP-1), studies have highlighted its roles in DNA repair, cellular stress mechanisms, and anti-aging benefits. Niacinamide was also studied for its antimicrobial activity, particularly in the context of host-infection via host immune response, yet its direct antimicrobial activity and the mechanisms of action remain unclear. Its multifunctionality makes it an appealing bioactive molecule for skincare products as well as a potential preservative solution. This study explores niacinamide’s antimicrobial mode of action against four common cosmetic pathogens. Our findings indicate that niacinamide is causing microbial cell cycle arrest; while cells were found to increase their volume and length under treatment to prepare for cell division, complete separation into two daughter cells was prevented. Fluorescence microscopy revealed expanded chromatin, alongside a decreased RNA expression of the DNA-binding protein gene, *dps*. Finally, niacinamide was found to directly interact with DNA, hindering successful amplification. These unprecedented findings allowed us to add a newly rationalized preservative facete to the wide range of niacinamide multi-functionality.

## 1. Introduction

Niacinamide (NAM) is an aromatic, water soluble carboxylic acid amide, which can be consumed from animal-sourced food products such as fish, poultry, meat, milk, and eggs, as well as in plants, seeds, and green vegetables. It is the amide form of niacin, which can also be endogenously synthesized in the liver from the essential amino acid tryptophan. Niacinamide is currently used extensively in cosmetic products due to its ample beneficial applications for skin health, including skin barrier and elasticity improvement, increased collagen and epidermal ceramide synthesis, reducing skin diseases such as hyperpigmentation, rosacea and acne, and improving overall skin appearance [1,2,3,4,5,6,7,8,9]. 

As the primary precursor of nicotinamide adenine dinucleotide (NAD+), an essential coenzyme in ATP production and the main substrate of the nuclear enzyme poly-ADP-ribose polymerase-1 (PARP-1), it has a very important role in redox reactions, energy processes, cellular metabolism, and mitochondrial function [8,10,11]. Therefore, niacinamide was investigated for its involvement in cellular stress response, DNA repair, and anti-cancerous mechanisms, where multiple health benefits were documented [12,13,14,15,16]. Although Niacinamide has been investigated for its multiple bioactive roles in humans since the 1970s, its antimicrobial role was discovered and investigated more thoroughly only later. The effect was demonstrated mainly in vivo, during host infection via host immune response and the production of various antimicrobial peptides (AMPs) by the host cells [17,18,19,20]. Preclinical data exist that demonstrate the tuberculostatic and antiretroviral effects of niacinamide [21,22,23]. The effect against *Mycobacterium tuberculosis* was explained with the inhibition of the class III NAD-dependent deacetylase-protein family, also called sirtuins [24], whereas the effect against HIV relied on the inhibition of the nuclear PARP-1 [22,25]. Moreover, niacinamide’s antibacterial activity strategically places it as an interesting active ingredient for acne treatment, as sustained by several studies [26,27,28,29].

Few studies suggested niacinamide to have a direct fungistatic effect. It was found to suppress the growth of several *Candida* and *Cryptococcus* spp. at relatively high concentrations, and to inhibit their biofilm formation, even in fluconazole resistant strains [30]. Confocal laser scanning microscopy revealed that niacinamide increased cell wall β-glucans exposure and chitin content with decreased mannan level. Moreover, *C. albicans* mutant lacking GIN4, which encodes a septin regulatory protein kinase and is essential for cell division and the maintenance of cell wall integrity, was identified to be highly sensitive to niacinamide, suggesting that niacinamide might exhibit antifungal activity by remodeling cell wall and affecting cell division regulatory processes. In another study, authors showed that niacinamide caused a decrease in *C. albicans*, *Trichophyton mentagrophytes*, and *T. rubrum* enzymatic activity of some esterase, esterase lipase, valin arylamidase, acid phosphatase, and α-glycosidase [31]. In a different study from the same year, it became clear that the decrease in enzymatic activity of *C. albicans* was related to Hst3p inhibition [32], a family member of the NAD+-dependent histone deacetylases family, sirtuins. 

Sirtuin enzymes utilize a two-step biochemical process to catalyze their deacetylation reaction, which allows for increased metabolic regulation via NAD+, by “sensing” the local environment for nutrient levels [33,34]. Acetylation can be divided into histone acetylation and non-histone, post translational protein acetylation. Histone deacetylation will allow chromatin condensation, thereby inhibiting gene transcription [35]. SIR2 inhibition by niacinamide will push the biochemical reaction towards more acetylated proteins that, in turn, will facilitate gene transcription. Putative homologs of sirtuins are encoded by bacteria, but only a few have been shown to function as true deacetylases, mostly in *Escherichia coli*, *Salmonella enterica*, and *Bacillus subtilis.* To date, only the regulation of a few bacterial sirtuin substrates has been characterized, while their metabolic roles are widely distributed, from DNA transcription, carbon and nitrogen metabolism, and protein translation through to virulence [36]. Regulation of transcription by reversible lysine acetylation has been widely studied in eukaryotes but not in bacteria. After the first bacterial acetylome studies, several transcription factors were suggested to be regulated by acetylation [37]. *E. coli* RcsB, a transcription factor involved in cell division and flagellum synthesis, was the first transcription factor identified as a sirtuin substrate in bacteria [38]. RcsB is a substrate of the deacetylase CobB, the most studied bacterial sirtuin, which was shown to be inhibited by niacinamide [39]. Sirtuins have emerged as important components of all regulation systems in bacteria, but the mechanism in which niacinamide inhibits bacterial sirtuins is far from being completely understood [40,41].

In this study, we explored the direct antimicrobial mechanism of action of niacinamide in various microorganisms, including Gram-negative and Gram-positive bacteria, yeast, and spore forming mold. These tested microorganisms are part of the pharmacopoeia list of microbes that are monitored in the final cosmetic products, as a contaminant risk for consumers. We aimed at elucidating the mode of action of niacinamide, as a desired molecule in the cosmetic market, which could also be beneficial in keeping final cosmetic applications free of contamination. We demonstrate that, consistent with a previous study [30], niacinamide inhibited microbial growth only at considerably high concentrations (MIC_100_ of 1.5–4%). Morphological examination via TEM images showed a significant effect on *C. albicans* cell wall and cell membrane (as previously demonstrated by fluorescence microscopy [30]). We also show an expansion of DNA material from the nucleolus into the cytoplasm following niacinamide treatment. Bacteria treated with niacinamide exhibited significantly large, elongated cells and a clear change in DNA spatial structure. *dps* (DNA-binding protein, a global regulator of gene expression, via chromatin reorganization [42]) gene expression levels, decreased gradually and significantly during niacinamide treatment, allowing the nucleoid to increase in size, resulting in DNA release and dispersal. Finally, we show that niacinamide directly interacts with DNA, causing DNA damage and interference with replications. This study paves the way to better understand niacinamide direct antimicrobial mode of action, which may allow the future development of a natural multifunctional preservative for skin care products. 

## 2. Materials and Methods

### 2.1. Microorganisms Strains and Growth Conditions

*Escherichia coli* (ATCC 8739), *Staphylococcus aureus* (ATCC 6538), *Pseudomonas aeruginosa* (ATCC 9027), *Candida albicans* (ATCC 10231), and *Aspergillus brasiliensis* (ATCC 16404) were obtained from ATCC and cultured according to the manufacturer’s instructions. These microorganisms are routinely used for the efficacy of preservatives in the cosmetic industry according to US and EU recommendations. Bacterial strains were maintained in tryptic soy agar (TSA) (HIMEDIA, Rehovot, Israel), while yeast and mold strains were maintained in sabouraud dextrose agar (SDA) (HIMEDIA, Rehovot, Israel), supplemented with oxytetracycline at a concentration of 1% (Hy-labs, Rehovot, Israel). *A. brasiliensis* was tested for antimicrobial efficacy and additional assays in this study at its spore form, as indicated by the USP 35, chapter <51> Antimicrobial effectiveness testing. 

### 2.2. Antimicrobial Activity (MIC and MBC)

The minimum inhibitory concentrations (MIC) of niacinamide were evaluated against tested microorganisms. Briefly, growth of *E. coli*, *S. aureus*, *P. aeruginosa*, *C. albicans*, and *A. brasiliensis* was evaluated following incubation with two-fold serial dilutions of niacinamide (Tianjin Zhongrui Pharmaceutical Co., Tianjin, China) in Mueller Hinton (MH) broth (HIMEDIA, Rehovot, Israel), for bacteria, and Sabouraud Dextrose broth (SDB) (HIMEDIA, Rehovot, Israel) for yeast and mold, using a 96-well plate (JET BIOFIL, Be’er Sheva, Israel). Wells were inoculated with 100 µL of test cultures with a final inoculum of 5 × 10^5^ CFU/mL of bacteria and 5 × 10^3^ CFU/mL of yeast and mold. Bacteria inoculated plates were incubated for 24 h at 32 °C, while yeast and mold inoculated plates were incubated for 48–72 h at 23 °C. Growth was evaluated by O.D._600_ read using TECAN Infinite^®^ 200 PRO microplate reader (TECAN, Kfar-Saba, Israel). To evaluate the minimum biocidal concentration (MBC) of niacinamide, 5 µL was taken from each well in the broth microdilution assay and used for seeding on top of TSA for bacteria or SDA for yeast and mold. In addition, 100 µL was taken from each well in the broth microdilution assay and were added in liquid Eugon broth (HIMEDIA, Rehovot, Israel) at a 1:1 dilution. Inoculated Eugon broth was incubated O.N. at 32 °C followed by O.D._600_ read. Data are presented as average value based on at least three independent experiments.

### 2.3. Transmission Electron Microscopy (TEM)

Test cultures of *E. coli*, *S. aureus*, *P. aeruginosa*, and *C. albicans* were treated with niacinamide at 0, 2.5, and 5%, and incubated for 4 h at 32 °C. Following treatment, cells were centrifuged at 3500× *g* for 10 min, supernatants were discarded, and pellets were transferred to Eppendorf tubes. Sample processing was performed as previously described [43]. Briefly, cells were re-suspended with a fixative, and further incubated for 2–3 h at R.T. Cells were then washed, post-fixed, and stained with 1% osmium tetroxide (Sigma-Aldrich, Jerusalem, Israel) and 1.5% potassium ferricyanide (Sigma-Aldrich, Jerusalem, Israel) in 0.1 M cacodylate buffer (Sigma-Aldrich, Jerusalem, Israel). Cells were washed again, followed by dehydration in increasing concentrations of ethanol, then washed again with 100% anhydrous ethanol. Then, cells were infiltrated with increasing concentrations of Agar 100 resin in ethanol. Cells were then embedded in fresh resin and incubated at 60 °C for 48 h. Embedded cells in blocks were sectioned with a diamond knife on a Leica Reichert UltraCut S microtome binocular microscope (Leica Microsystems, Wetzlar, Germany). Ultrathin sections (80 nm) were collected onto a 200 Mesh thin bar copper grids (Sigma-Aldrich, Jerusalem, Israel). The sections on grids were sequentially stained with uranyl acetate and lead citrate for 10 min each time and viewed using Tecnai 12 TEM 120 kV (Phillips, Eindhoven, The Netherlands) equipped with a Phurona camera and RADIUS software version 2.3 (Emsis GmbH, Münster, Germany). For *C. albicans*, samples were centrifuged for 2 min at 3000× *g*, supernatants were discarded, and pellets were transferred to Eppendorf tubes. Cells were re-suspended in 0.5 mL 1.5% KMnO_4_ (*w*/*v* in ddH_2_O) and incubated for 1 h at room temperature. Samples were centrifuged 1 min at 3000× *g* and supernatants were discarded. The samples were rinsed several times in ddH_2_O until samples medium stayed clear. Next, the dehydration process in ethanol was conducted as mentioned above. This part of the work was performed by the Bio-imaging Unit of The Hebrew University in Jerusalem (Dr. Yael Friedmann) and by the Institute of Nanotechnology and Advanced Materials at Bar-Ilan University in Ramat Gan (Sabanay Helena MA).

### 2.4. Phase Microscopy and Image Analysis

Phase contrast images of *E. coli*, *S. aureus*, *P. aeruginosa*, and *C. albicans* were taken by using an ECLIPSE Ti2 (Nikon, Tokyo, Japan), equipped with a Prime BSI camera (Photometrics, Roper Scientific, Tucson, AZ, USA). Cell size analysis was performed manually by using Fiji software version 2.8.2. By adjusting the threshold (0–105), the cells’ areas were marked. Adjacent cells were separated by using Watershed function followed by size analysis limited to the range of 0.1–10 µm^2^. In addition, cell count analysis was derived from the same cell size image analysis. Counting the dividing cells was performed manually by counting only the cells with distinguishable septum.

### 2.5. Fluorescence Microscopy and Image Analysis

Test cultures of *E. coli*, *S. aureus*, *P. aeruginosa*, and *C. albicans* were treated with niacinamide at 0, 2.5, and 5% for 4 h at 32 °C. Following incubation, 3 mL of cultures were centrifuged at 8600× *g* for 3 min and pellets were washed three times with DPBS ×1 (Kibbutz Beit-Haemek, Israel). Subsequently, pellets were resuspended in PBS ×1 supplemented with 10 µg/mL DAPI dye (Invitrogen, Carlsbad, CA, USA), loaded on microscope slides and photographed using an ECLIPSE Ti2 (Nikon, Tokyo, Japan), equipped with Prime BSI camera (Photometrics, Roper Scientific, Tucson, AZ, USA). For DAPI stain imaging filter sets pE-800, Line 1, ExW: 400 excitation and wheel1-MXR00714—LED-DAPI-A (DAPI) (FF02-447/60) (Nikon, Tokyo, Japan), emission was used. System control performed by using NIS-Elements AR Analysis (version 5.30.05; Nikon, Tokyo, Japan). DAPI area analysis was performed manually using Fiji software (version 2.8.2); by adjusting the threshold (100–225), the DAPI area was marked, followed by a size analysis limited to the range of 0.1–8 µm^2^. To calculate the percentage of DAPI area per total microbial cell area, total DAPI area was divided by the total area of the cells calculated from the counter phase images and multiplied by 100. 

### 2.6. DNA-Binding Protein (dps) Expression Levels

*E. coli* culture was incubated O.N. and then diluted to O.D._600_ of 0.05, and further incubated for 2 h to reach the early logarithmic phase. Then cultures were incubated with 0, 1, 2.5, and 5% niacinamide for 1, 3, and 24 h at 32 °C with shaking (180 rpm). RNA was extracted from the cells by using GeneJET RNA Purification Kit (ThermoFisher Scientific, Modi’in, Israel). Then, 1 µg of RNA was reversed transcribed using High-Capacity RNA-to-cDNA™ Kit (ThermoFisher Scientific, Modi’in, Israel). qPCR reaction was performed using *16S* as the housekeeping gene with a forward primer 519R (5′-GWATTACCGCGGCKGCTG-3′ and a reverse primer 341F 5′-CCTACGGGDGGCWGCA-3′), while the target *dps* gene was used with forward primer 5′-GCTTTATACCCGCAACGATG-3′ and a reverse primer 5′-CCATCCAGCATTTCATGTACG-3′ (Hy-labs, Rehovot, Israel). Primers’ efficiency was verified via standard curves quantification using serial dilutions. qPCR reactions were performed in triplicates using SsoAdvanced Universal SYBR Green Supermix (Bio-Rad Laboratories, Haifa, Israel) with the CFX 96 instrument (Bio-Rad, Haifa, Israel). Cycling conditions were 95 °C for 3 min, then 39 cycles of 95 °C for 10 s, 55° C for 30 s, followed by a melt curve analysis. Results of Cq from target gene *dps* were normalized against the mRNA expression of the reference gene *16S* rRNA in each cDNA sample. Data are presented as normalized values of 2^−∆∆CT^, relative to the control treatment (0% NAM at 1 h).

### 2.7. Niacinamide Direct Interaction with DNA

An 870 bp amplicon was amplified from *gyrB* target gene using DreamTaq PCR Master Mix (×2) (ThermoFisher Scientific, Modi’in, Israel) according to the manufacturer’s instructions. PCR reaction was performed using CFX 96 instrument (Bio-Rad, Haifa, Israel) with cycling parameters of 1 min at 95 °C, then 40 cycles of 95 °C for 30 s, 50 °C for 30 s, and 72 °C for 1 min, followed by a final elongation step at 72 °C for 10 min. PCR product was cleaned using GeneJET PCR Purification Kit (ThermoFisher Scientific, Modi’in, Israel) while DNA concentration was determined using a NanoDrop Lite Plus Spectrophotometers (Thermo Scientific™, Modi’in, Israel). Then, 80 ng of DNA was incubated for 2 h at room temperature (R.T.) with increasing concentrations of niacinamide at 0, 5, 10, 20, and 30% (diluted in ddH_2_O). Samples were then loaded onto a 2% EX E-gel (ThermoFisher Scientific, Modi’in, Israel), with GeneRuler 50 bp DNA Ladder (Thermo Scientific™, Modi’in, Israel), and ran according to the manufacturer’s instructions. A positive control of denatured DNA, heated at 95 °C for 10 s, was used. Then, qPCR amplification of the same 870 bp DNA fragment was conducted in the presence of 0, 5, 10, and 15% niacinamide, using SsoAdvanced Universal SYBR Green Supermix (Bio-Rad Laboratories, Haifa, Israel), according to the manufacturer’s instructions. Cycling conditions were 95 °C for 3 min, then 39 cycles of 95 °C for 10 s, 55° C for 30 s, followed by a melt curve analysis. DNA concentrations of the clean PCR products were determined, and the samples were loaded onto a 2% EX E-gels.

### 2.8. Statistical Analysis

Statistical analysis of the data was performed using Prism GraphPad version 10.2.3 (GraphPad Software, 2024). Quantitative analysis of the bacterial cell volume was statistically analyzed using one-way ANOVA with Tukey’s multiple comparisons as a post test. Quantitative analysis of the microscopic fluorescence images DNA content from the total cell area (Figure S6) was analyzed separately for each microorganism using one-way ANOVA with Tukey’s multiple comparisons as a post test. Phase images microscopic analysis of the microorganisms cell count and percentages of dividing cells, as well as *dps* gene expression levels, were statistically analyzed using two-way ANOVA with Tukey’s multiple comparisons as a post test. Data are presented as mean values ± SD.

## 3. Results and Discussion

### 3.1. Antimicrobial Activity via MIC and MBC

The antimicrobial activity of niacinamide against the five pharmacopoeia strains (ISO 11930 (https://www.iso.org/standard/75058.html, accessed on 7 July 2024)) was evaluated using MIC_100_ and MBC_100_ (Table 1). It was demonstrated that only at high concentrations of ≥15,000 ppm (1.5%), niacinamide reduced or inhibited microbial growth. However, the concentration required to kill the microorganisms (MBC_100_) was significantly higher at ≥60,000 ppm (6%). Therefore, to achieve sufficient efficacy, by using an additional antimicrobial compound in a skin care formulation, niacinamide broad-spectrum antimicrobial mechanism of action should be further elucidated [44]. Specifically, niacinamide was most active against Gram-negative *P. aeruginosa*, an opportunistic pathogen, well known for its ability to survive and resist extreme environments and antimicrobial assault [45]. 

### 3.2. Morphological Effect of Niacinamide Using TEM

**Figure 1:**
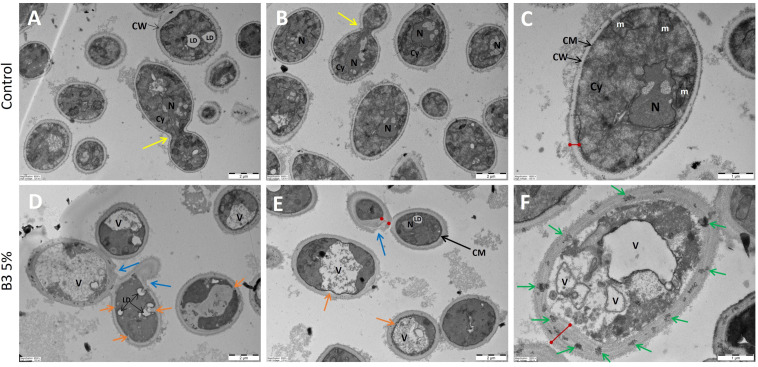
*C. albicans* TEM image of control and niacinamide treated cells. (**A**–**C**) Control cells. (**D**–**F**) 5% Niacinamide treatment. N = nucleus, CM = cell membrane, CW = cell wall, V = vacuole, LD = lipid droplet, m = mitochondrion, Cy = cytoplasm, red lines = cell wall thickness, yellow arrows = budding cell, blue arrows = external cell wall modifications, green arrows = cell wall condensed material, orange arrows = invagination of cell membrane and cell wall. (**A**,**B**,**D**,**E**) have a 2 µm scale bar, (**C**,**F**) have a 1 µm scale bar.

**Figure 2:**
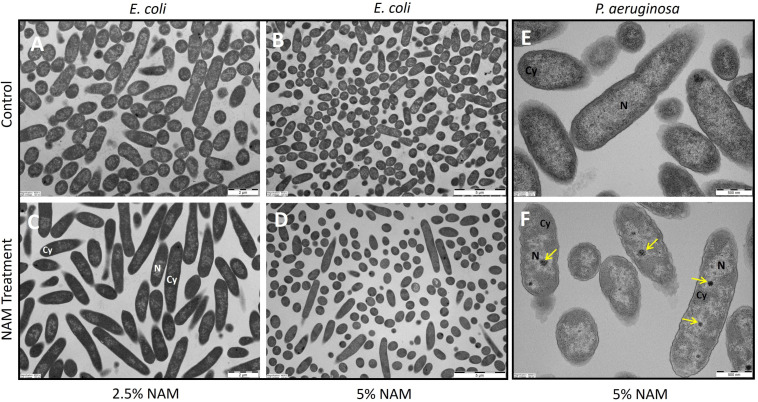
TEM images of control and niacinamide treated cells. (**A**,**B**) *E. coli* control cells. (**C**) *E. coli* under 2.5% Niacinamide treatment. (**D**) *E. coli* under 5% Niacinamide treatment. (**E**) *P. aeruginosa* control cells. (**F**) *P. aeruginosa* under 5% Niacinamide treatment N = nucleoid, Cy = cytoplasm, yellow arrows = dense granules. (**A**,**C**) have a 2 µm scale bar, (**B**,**D**) have a 5 µm scale bar and (**E**,**F**) have a 500 nm scale bar.

**Figure 3:**
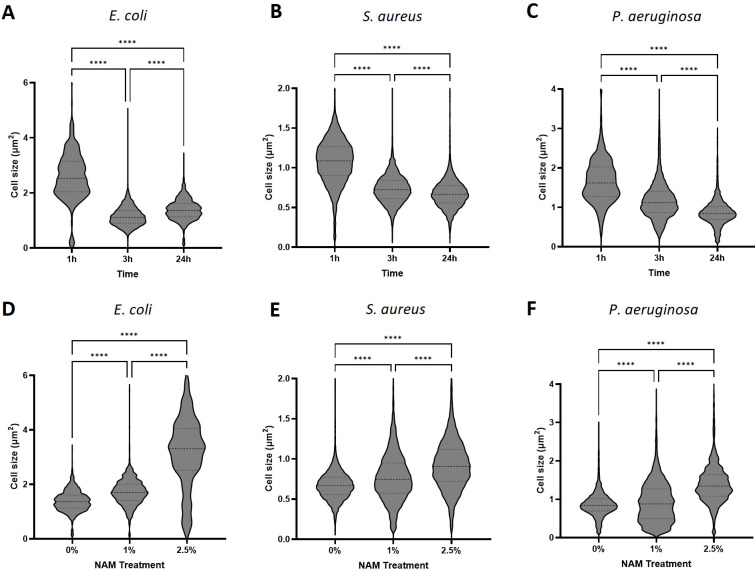
Bacterial cell size analysis from microscopic phase images. Control treatment of microorganisms incubated in TSB medium of (**A**) *E. coli*, (**B**) *S. aureus* and (**C**) *P. aeruginosa*. Niacinamide treatment at concentrations of 0, 1 and 2.5% following 24 h incubation of (**D**) *E. coli*, (**E**) *S. aureus* and (**F**) *P. aeruginosa*. Corresponding phase images are located in Supplementary Figures S2–S5. (**** = *p* value < 0.0001).

Initially, morphological changes due to niacinamide treatment were examined using TEM images of tested microorganisms. Niacinamide treatment appears to have a significant effect on *C. albicans* cell wall and cell membrane (Figure 1). However, in other tested microorganisms, the cell wall and cell membrane appear to be either slightly or not effected at all (Figure 2, Figure 3 and Figure S1).

In *C. albicans* control treatment images (Figure 1A–C), cells appear healthy and intact, while intracellular organelles and nucleus appear clearly. Niacinamide treatment at 5% resulted in significant thickening of the cell wall (red line), with various cell wall modifications (blue, orange, and green arrows). In some cases, the cell membrane lost its oval shape (Figure 1E, orange arrows), and invagination of the cell wall was observed (Figure 1D, orange arrows). Cell wall components appear leaking into the cytoplasm, aggregating in lipid droplets (LD). The number of vacuoles increased significantly, and the nucleus no longer seems intact and organized. Aligned with our results, indicating an increase in cell wall size, in a previous study by XinRui Xing et al., confocal laser scanning microscopy images of niacinamide treated cells show a significant increase in the fluorescence signal of monoclonal β-1,3-glucan antibody in comparison to the control. In addition, Calcofluor White (CFW) staining revealed that chitin content increased as well, while mannan content, indicated by ConA staining, decreased [30].

As opposed to the *C. albicans* TEM images, in *S. aureus*, morphological changes were less significant (Figure S1). In *P. aeruginosa* treated cells, an electron dense material was observed (Figure 2F, yellow arrows). According to the literature, these electron dense granules might be polyphosphate granules, which are formed by the consumption of ATP, as part of a conserved bacterial starvation survival response. In *P. aeruginosa*, polyphosphate granules biogenesis was tied to nitrogen starvation and cell cycle exit [46]. Being an essential co-enzyme in ATP production, it suggests that niacinamide treatment caused an increase in ATP levels, which subsequently induced its consumption, resulting in polyphosphate granules presence. Niacinamide effect on *E. coli* cells, at a concentration of 2.5%, mainly resulted in significantly large, elongated cells, around 5 µm long (Figure 2C). At 5% niacinamide (Figure 2D), most cells already decreased their volume back, also having a spherical shape; however, some of the cells continued to appear elongated. From the same study by XinRui Xing et al., *C. albicans* cells appeared extremely enlarged [30], an observation which was not seen in our images. However, the concentration used in their study was 20 times lower than the concentration used in ours. 

### 3.3. Phase Microscopy Image Analysis

#### 3.3.1. Cell Volume

The significant increase in *E. coli* cell length and size, observed by the TEM images, led us to quantify all tested microorganisms’ cell size, using phase microscopy images, followed by image analysis using Fiji. In the no treatment control cultures, during incubation in TSB growth medium, all bacteria were shown to significantly reduce their cell size following 3 and 24 h incubation times, indicated by both quantitative analysis and the phase images (quantitative analysis: *p* < 0.0001. Figure 3A–C and Figure S2). Bacteria in their stationary phase are known to undergo adaptation of morphology, which is critical for survival; thus, the size of most bacteria is considerably reduced upon entry into stationary phase [47,48,49,50]. According to Lange and Hengge-Aronis, sigma factor σ^38^ encoded by the *rpoS* gene in *E. coli* was found to be an important regulator, in which its expression levels are upregulated during the entry of the bacteria to their stationary phase, observed by smaller volume spherical shape cells. Transition to σ^38^ influences not only metabolic and regulatory pathways, but also dramatically alters the physiology of the bacterial cell. Consistent with the literature, phase images of Gram-negative *E. coli* and *P. aeruginosa* lost their rod shape and look smaller and spherical. In addition, bacterial cells in their stationary phase have an increased cell envelope resilience, increase in the level of cross-linking in the peptidoglycan, and an increase in lipoprotein content [51,52,53]. In addition, stationary-phase *E. coli* cells are resistant to heat, H_2_O_2_, and high osmolarity [54,55], as the cells activate the stringent response mechanism to survive nutrient deficiency, starvation, and stressful environmental conditions. 

During treatment with niacinamide, all three microorganisms tested show a significant increase in cell size (Figure 3D–F and Figures S3–S5), specifically in higher niacinamide concentrations of 2.5% and following longer incubation time (24 h). In *S. aureus* and *P. aeruginosa*, this effect was seen only at 3 and 24 h incubation. In *E. coli*, cells increased their size significantly starting from 1 h incubation time (Figure S3A). The maximum average increase in cell size was most significant in *E. coli* (∆1.8 µm^2^), followed by *P. aeruginosa* (∆0.53 µm^2^), and finally the least in *S. aureus* (∆0.25 µm^2^). The effect of reduction in cell volume during stationary phase to sustain environmental stress was attenuated, suggesting that niacinamide treatment can leave the cells susceptible to any damaging factors in the environment, and may be used as a booster in an antimicrobial mixture. 

#### 3.3.2. Cell Count and Percentage of Dividing Cells

**Figure 4:**
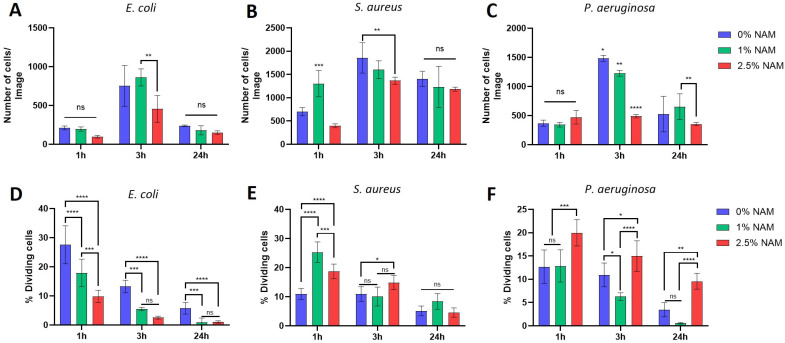
Microscopic analysis of the microorganisms’ phase images. Cell number and percentage of dividing cells are presented under niacinamide treatment at 0, 1 and 2.5% at incubation time of 1, 3, and 24 h. The number of counted bacterial cells per image (**A**) *E. coli*, (**B**) *S. aureus*, and (**C**) *P. aeruginosa*. The number of dividing cells as a percentage from the total number of cells (**D**) *E. coli*, (**E**) *S. aureus* and (**F**) *P. aeruginosa*. (**** = *p* value < 0.0001, *** = *p* value < 0.001, ** = *p* value < 0.01, * = *p* value < 0.05, ns = not significant).

Further analysis of the microorganisms’ cell count from the same phase images revealed an expected general trend in the non-treated cells (Figure 4A–C, blue bars), in which the number of cells increases after 3 h incubation due to multiple cell divisions, followed by a decrease in number of cells at 24 h incubation, due to the cells entering their death phase. Treatment with niacinamide at 2.5% caused a decrease in cell count, mainly following 3 h incubation time, in all bacteria. When observing the percentage of dividing cells from these images (Figure 4D–F), it was shown that *E. coli* had a niacinamide-concentration dependent effect of reduced divisions (Figure 4D). In *S. aureus*, which is the most resistant microorganism to niacinamide treatment (Table 1; MIC_100_), there was an overall increase in number of dividing cells due to niacinamide treatment (Figure 4E). *P. aeruginosa*, which is the most sensitive to niacinamide (Table 1; MIC_100_), also had an increase in cell division at 2.5% niacinamide (Figure 4F). While cell count was decreased during niacinamide treatment in all microorganisms, and most significantly in *P. aeruginosa* (Figure 4C, 3 h incubation at 2.5% niacinamide, *p* value < 0.0001), the percentage of dividing cells is decreased in *E. coli*, but rather increased significantly in *S. aureus* and *P. aeruginosa*. It appears that under niacinamide treatment, all tested microorganisms are prepared for division by various mechanisms, such as a significant elongation of the cells (*E. coli*) and increased cell. In contrast with *E. coli*, the septum formation initiated during treatment with niacinamide is induced in *S. aureus* and *P. aeruginosa*, although separation into two daughter cells does not occur.

### 3.4. Fluorescence Microscopy Image Analysis

Microorganisms are known to regulate their cell cycle by coordinating the cell division with DNA replication status. Therefore, to study the effect of niacinamide on microbial cell cycle, DNA staining was performed to visualize the content, structure, and location of the DNA [56,57] under niacinamide treatment.

**Figure 5:**
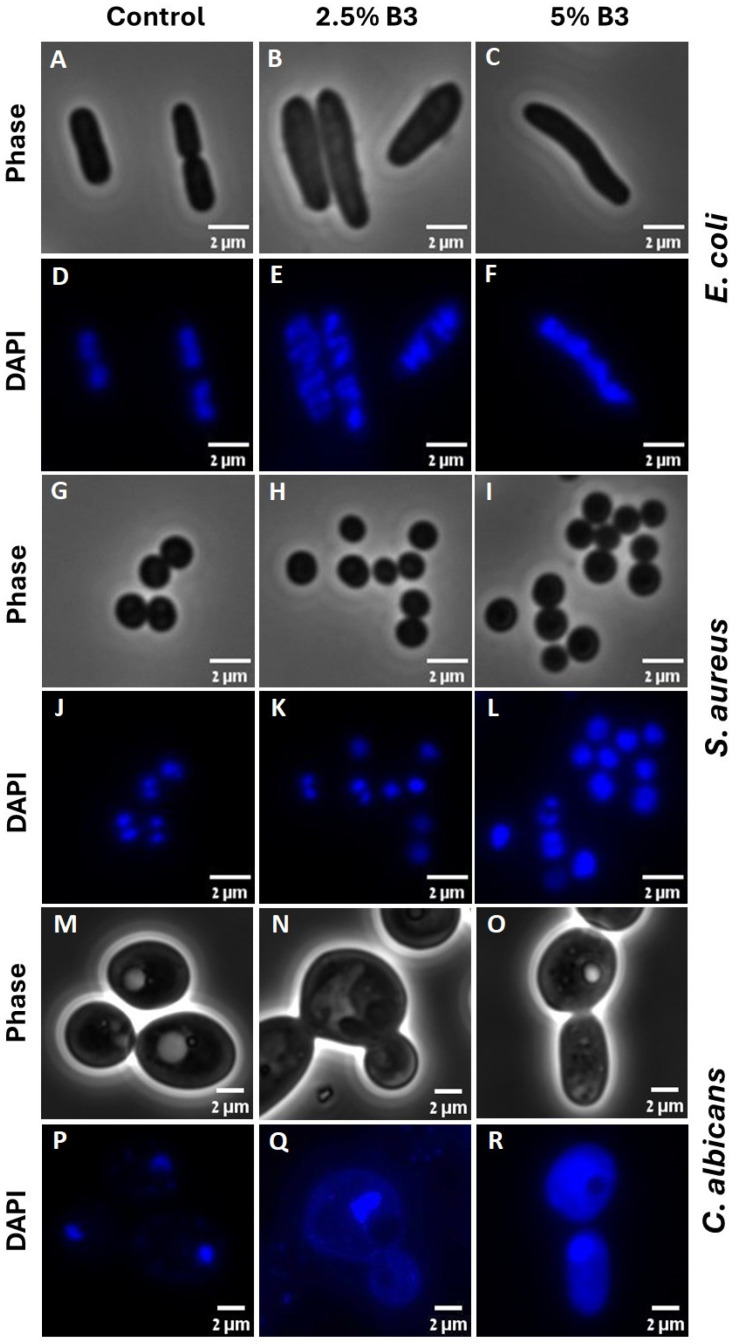
Fluorescence images of DAPI staining, along with the comparative phase image in *E. coli* (**A**–**F**), *S. aureus* (**G**–**L**) and *C. albicans* (**M**–**R**), followed by 4 h treatment with either 2.5 or 5% of niacinamide.

DAPI staining of control cells showed a normal chromosome morphology (Figure 5D,J,P), with either one or two discrete nucleoids (nuclei in *C. albicans*), with a septum formation visible (phase images of *E. coli* and *S. aureus*, Figure 5A and Figure 5G respectively). With the increasing concentrations of niacinamide, DNA stain was dispersed throughout the cells, occupying a larger volume of the cell. In *E. coli*, binucleate cells show no septum (Figure 5B,E). In *C. albicans*, at 2.5% niacinamide, mitosis occurred without a clear separation of the nuclei (Figure 5N,Q). These fluorescence images are in concordance with our previous data indicating that under niacinamide treatment, the microbial cells are preparing for division, visible by enlarged nucleoid/nucleus, although division is arrested at some point during the cycle. Further analysis of the DNA content from the fluorescence images was performed (Figure S6), where the DNA fluorescence staining area was calculated as a percentage from the entire cell area using Fiji software. While a general trend of increasing DNA content was seen with the increasing niacinamide concentrations, a significant effect of DNA dispersal was seen at 5% niacinamide in comparison to the control in all microorganisms. Of note, DAPI staining was not suitable for *P. aeruginosa* and *A. brasiliensis* spores, as fluorescent images obtained were of poor quality.

In *C. albicans*, the nucleus structure is maintained by the nuclear envelope, the nuclear pore complex, and the nucleolus. SIRT2 is known to regulate nuclear envelope reassembly during DNA replication and cell cycle, while SIRT2 depletion caused defects in envelope structure [58]. As niacinamide is known to be a SIR2 inhibitor, treatment might subsequently cause leakage of DNA material from the nucleolus to the cytoplasmatic area. In bacteria, niacinamide is known also to inhibit sirtuin-like enzymes responsible for post-translational deacetylation, shown by in vitro inhibition of CobB, a lysine delactylase involved in metabolism regulation [39]. Here, we show indications that niacinamide treatment is causing dis-organization and dis-localization of the bacterial and yeast DNA, visibly detected by DNA staining and fluorescence images, due to probable malfunction of some DNA-associated proteins and nuclear envelope [41,59].

### 3.5. dps Gene Expression

There are several mechanisms in which PARP-1 and NAD^+^-dependent reactions can directly control chromatin structure, subsequently effecting genomic stability, cell division and differentiation, and apoptosis in eukaryotes. Indeed, it was implied that niacin status can cause chromatin relaxation, control of cell cycle arrest, and apoptosis [60,61,62]. In bacteria, there are a multitude of nucleoid-associated proteins responsible for maintaining the nucleoid structure, chromosome packaging, and transcription, whose expression depends on the growth phase of the bacterial culture. The IHF, HU, *dps*, Fis, and H-NS proteins are considered the most essential structural proteins of the nucleoid [63]. In the bacterial stationary phase, the nucleoid becomes more condensed to protect the DNA from damage. This mechanism is mostly governed by *dps* protein (DNA-binding protein from starved cells), a ferritin-like iron-binding protein, which is capable of non-specific binding to the DNA, and is active exactly in periods of starvation [63,64]. In addition, *dps* gene expression levels could be triggered at other growth stages due to oxidative stress, or other various environmental insults that can damage the DNA [64,65]. 

**Figure 6:**
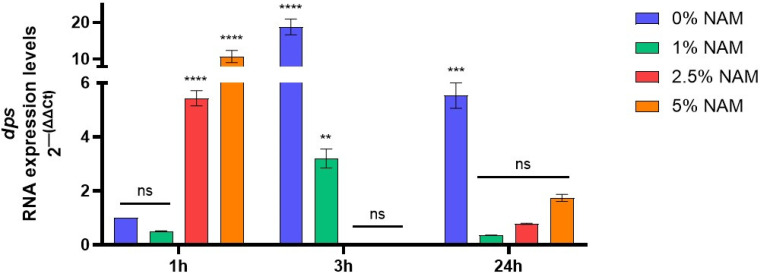
*E. coli dps* RNA expression levels during incubation with 0–5% niacinamide, at various cell cycle stages, logarithmic phase of 1 h, stationary phase of 3 h and following 24 h. *dps* gene expression was assessed against the 16S rRNA housekeeping gene. Data are presented as 2^−ΔΔCT^ normalized expression levels to the 0% negative control treatment at 1 h, ± SD. Significant differences are indicated in the graph (*p*-value **** < 0.0001, *** < 0.001, ** < 0.01, ns = not significant).

We then measured the RNA expression levels in *E. coli* during treatment with niacinamide at various growth phases. We found that in the control treatment, *dps* gene expression levels increased at 3 h incubation time (Figure 6), indicating the entrance into the stationary phase, also supported by the decreased cell volume and rod shape bacteria turning spherical. However, under niacinamide treatment at 3 h incubation time, *dps* expression levels decreased gradually and significantly, which might allow the nucleoid to increase its size, resulting in DNA release, unfolding, and dispersal. At 1 h incubation time, *dps* expression levels under 2.5–5% niacinamide show an opposite effect with significant upregulation, perhaps due to oxidative stress caused by the treatment. 

### 3.6. Interaction with DNA Fragment

From our data so far, we concluded that niacinamide is causing increase in cell size and length, DNA release and nucleoid-associated protein remodeling, theoretically allowing for DNA replication. These cellular activities are driving the microbial cell to prepare for division into two daughter cells (mitosis in *C. albicans*); however, cell division is arrested. We wanted to verify whether niacinamide is causing cell cycle arrest only via interaction with the nucleoid associated/regulatory proteins/enzymes, or whether it can also directly intercalate with the DNA, causing DNA damage and interference with replications. For this purpose, an 870 bp DNA fragment was incubated with increasing concentrations of niacinamide and then ran on electrophoresis gel to observe migration of the amplicon based on size or conformational changes of the dsDNA, as previously performed by Srivastava et al. [66].

**Figure 7:**
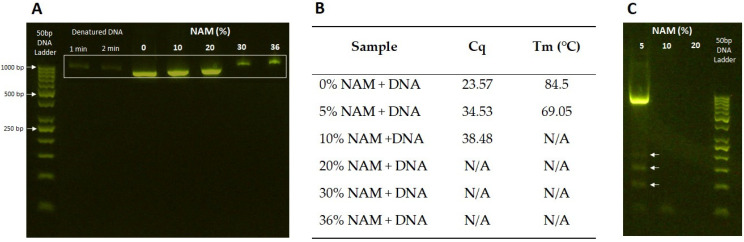
Niacinamide interaction with DNA amplicon by gel electrophoresis and qPCR. (**A**) Agarose gel image of DNA fragment incubated with niacinamide at increasing concentrations. (**B**) qPCR assay results (Cq = cycle of amplification, Tm = melting temperature of the amplicon) with amplicon’s relevant primers. (**C**) Clean PCR products of amplified DNA treated with increasing concentrations of niacinamide (5–20%), arrows represent fragmented DNA product.

A clear shift in DNA migration was observed on 2% agarose gel (Figure 7A), starting from a slightly slower migration at 10–20% up to significant shift at 30–36% niacinamide. Then a qPCR assay of DNA incubated with niacinamide showed that at 5% niacinamide, the cycle threshold for amplification initiation increases, while the melting temperature (Tm) is decreased, indicating a shorter amplicon size (Figure 7B). At concentrations > 5% niacinamide there was no successful amplification observed. Then, PCR products from the same qPCR assay were cleaned to remove the primers and any residual niacinamide molecules and were run on an agarose gel once again. At 5% niacinamide treatment, where there was a clear amplification of the DNA, one bright bend at the right size was visible; however, fragmentation of the DNA, observed by smaller size bends, were visible on the gel (Figure 7C, white arrows), indicating a failure to successfully amplify the DNA. These results suggest that niacinamide can bind the DNA itself, causing interference with DNA replication. Small molecules binding to dsDNA through either intercalation or minor groove binding can alter its structure and mechanical properties [67]. Variously known antimicrobial and antiparasitic small molecules with a minor groove binding mechanism were previously studied, while intercalation is known to lengthen and unwind the DNA helix, while minor groove binding has only a minor to no effect on DNA length and winding angle [68,69,70,71].

## 4. Conclusions

There are numerous studies conducted on niacinamide’s multiple therapeutic functions and implications. One major attribute of this molecule is its antimicrobial activity, which was investigated in the context of host infection, coordinated by the host immune response, and the production of antimicrobial peptides (AMPs). Yet, its direct antimicrobial activity was believed to be ineffective to inhibit and kill bacteria [8]. Direct antifungal activity of niacinamide against several *Candida* spp. and *Cryptococcus neoformans* was demonstrated by MIC_50_ and reached 20–80 mM (0.25–1%) [30]. In this study, we investigated the antibacterial and antifungal activity of niacinamide using MIC_100_ and MBC. MIC_100_ was found to be in the range of 1.5–4%, with *P. aeruginosa* being the most sensitive to treatment and *A. brasiliensis* being the most resistant. MBC assay revealed that niacinamide bactericidal and fungicidal activities were at a concentration of ≥6% (~0.5 M), with *A. brasiliensis* and *S. aureus* being the most resistant microorganisms. Further investigation of niacinamide direct antimicrobial mode of action using TEM and phase microscopy images revealed that *E. coli*, *S. aureus*, and *P. aeruginosa* increased their cell volume significantly under treatment. Gram-negative rod shape bacteria were elongated, and *P. aeruginosa* and *S. aureus*, but not *E. coli*, had visible septum, as the cells were preparing to divide into two daughter cells; however, all microorganisms failed to complete the division process. DNA staining showed that niacinamide treatment caused DNA material to cover larger areas of the cells and combined with decreased gene expression levels of the DNA-binding protein gene, *dps*, suggesting induced chromatin relaxation and DNA replication, once again as a preparation for cell division. Finally, we showed that niacinamide can directly bind to DNA fragments, probably interfering with DNA replication causing fragmented DNA amplicons. This study highlights niacinamide potential antimicrobial mode of actions to allow its use as a multifunctional antimicrobial agent and a skin bioactive molecule in personal care products. 

## Figures and Tables

**Table 1:** MIC_100_ and MBC_100_ of niacinamide against various microorganisms represented in ppm.

	Niacinamide (ppm)	
Microorganism	MIC_100_	MBC_100_
*E. coli*	30,000	60,000
*S. aureus*	25,000	>60,000
*P. aeruginosa*	15,000	60,000
*C. albicans*	30,000	60,000
*A. brasiliensis*	40,000	>60,000

## Data Availability

The original contributions presented in the study are included in the article/supplementary material, further inquiries can be directed to the corresponding author.

## Supplementary Materials

The following supporting information can be downloaded at: https://www.mdpi.com/article/10.3390/microorganisms12081581/s1, Figure S1: *S. aureus* TEM image of control and niacinamide treated cells. Figure S2: Bacterial cell size analysis following incubation in TSB. Figure S3: *E. coli* cell size analysis during incubation with 0, 1 and 2.5% niacinamide. Figure S4: *S. aureus* cell size analysis during incubation with 0, 1 and 2.5% niacinamide. Figure S5: *P. aeruginosa* cell size analysis during incubation with 0, 1 and 2.5% niacinamide. Figure S6: Quantitative analysis of DNA content by calculating DAPI staining area proportion out of the entire microbial cell area. Figure S7: Original gel image. Niacinamide interaction with DNA amplicon by gel electrophoresis and qPCR. Agarose gel image of DNA fragment incubated with niacinamide at increasing concentrations. Figure S8: Original gel image. Clean PCR products of amplified DNA treated with increasing concentrations of niacinamide.
