# Supplementary material for: Niacinamide Antimicrobial Efficacy and Its Mode of Action via Microbial Cell Cycle Arrest
Source: Microorganisms. 2024 Aug 2;12(8):1581. doi: 10.3390/microorganisms12081581 (PMC11356291; doi:10.3390/microorganisms12081581)
Supplement: Supplementary file 1 [file microorganisms-12-01581-s001.zip › microorganisms-3120093-supplementary.pdf]

microorganisms

MDPI

Supplementary material

Niacinamide Antimicrobial Efficacy and its Mode of Action *via* Microbial Cell Cycle Arrest

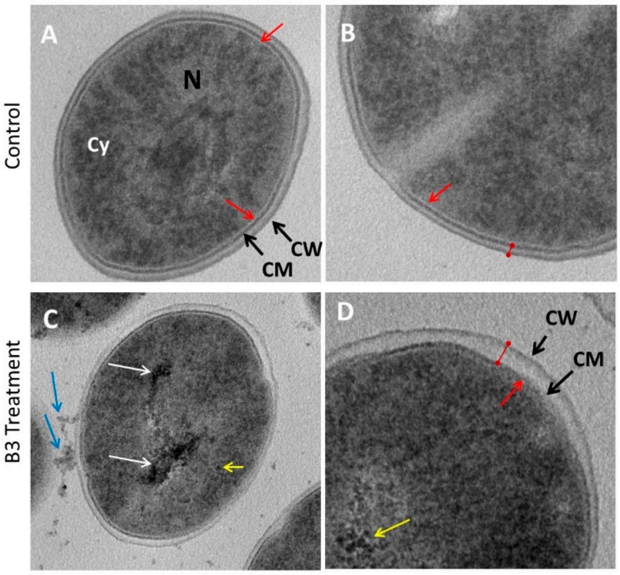

Figure S1. *S. aureus* TEM image of control and niacinamide treated cells. (**A-B**) Control cells. (**C-D**) 5% Niacinamide treatment. N = nucleoid, Cy = cytoplasm, CW = cell wall, CM = cell membrane, red lines = cell membrane size, white arrows= condensed nucleoid proteins, yellow arrows = dense granules, blue arrow = cell debris.

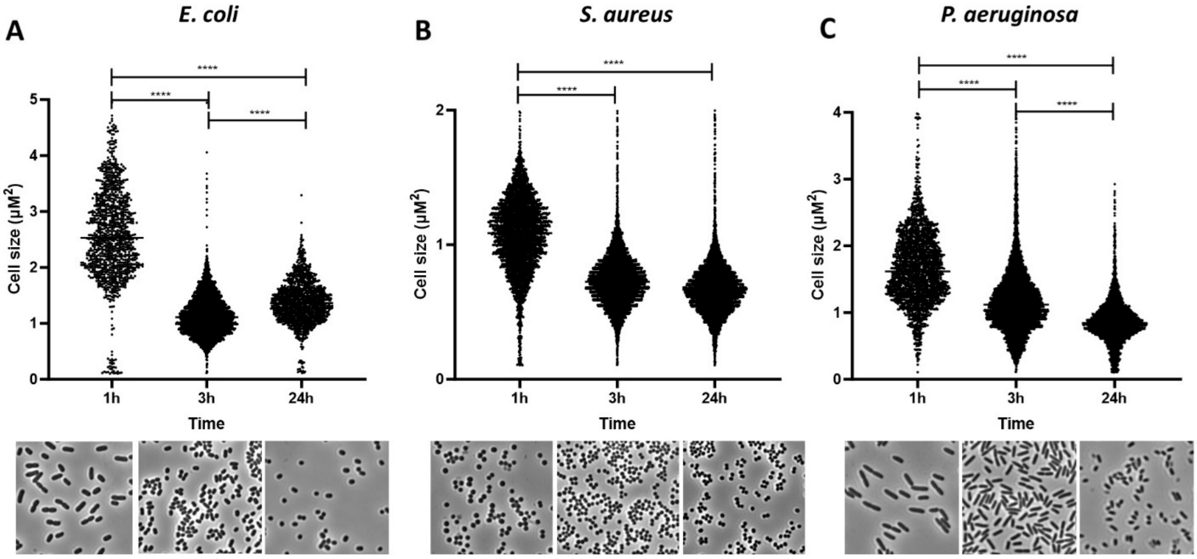

Figure S2. Bacterial cell size analysis following incubation in TSB. Phase images were taken at 1, 3, and 24 hours and are located under the graphical measurements accordingly. (**A**) *E. coli*, (**B**) *S. aureus* and (**C**) *P. aeruginosa*. (****=*p* value < 0.0001)

microorganisms

MDPI

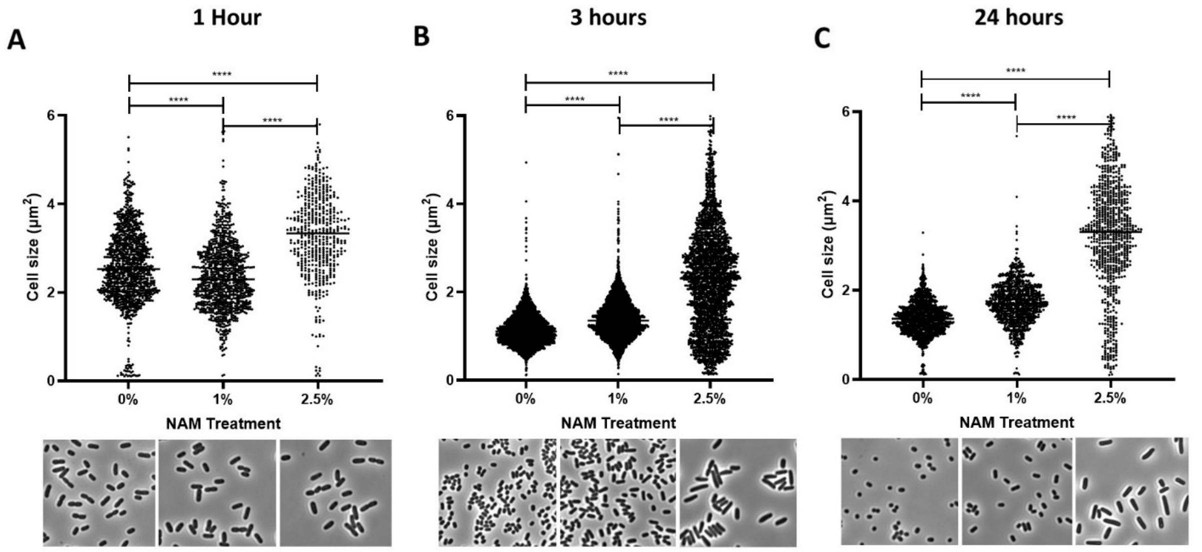

Figure S3. *E. coli* cell size analysis during incubation with 0, 1 and 2.5% niacinamide. Incubation periods were (**A**) 1h, (**B**) 3h and (**C**) 24h. Phase images of *E. coli* under niacinamide treatment at concentrations of 0, 1, 2.5% are located under the graphical measurements accordingly. (****=p value < 0.0001).

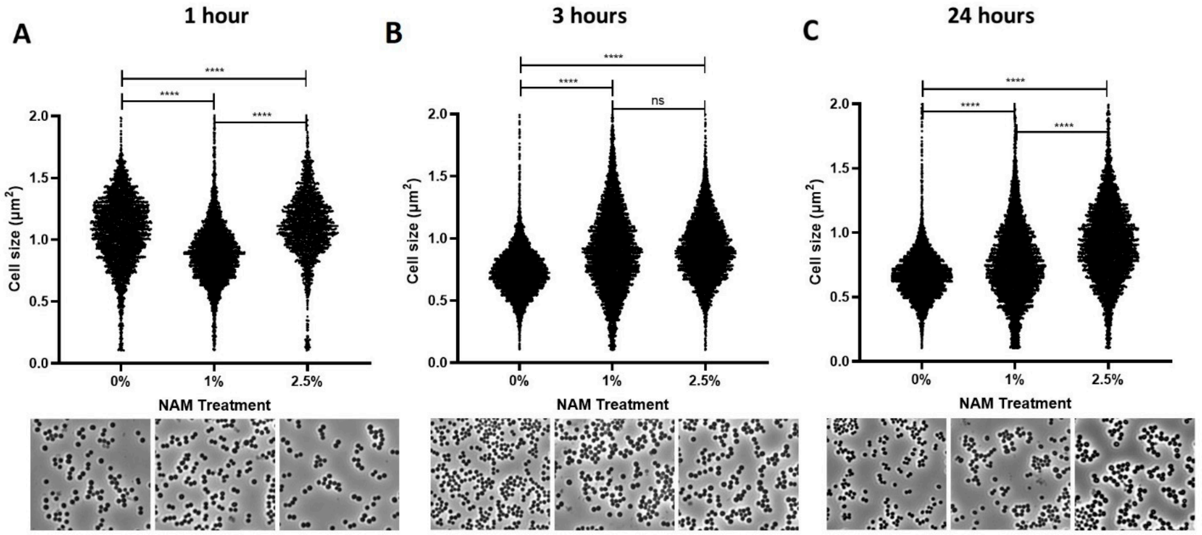

Figure S4. *S. aureus* cell size analysis during incubation with 0, 1 and 2.5% niacinamide. Incubation periods were (**A**) 1h, (**B**) 3h and (**C**) 24h. Phase images of *S. aureus* under niacinamide treatment at concentrations of 0, 1, 2.5% are located under the graphical measurements accordingly. (****=p value < 0.0001, ns= not significant).

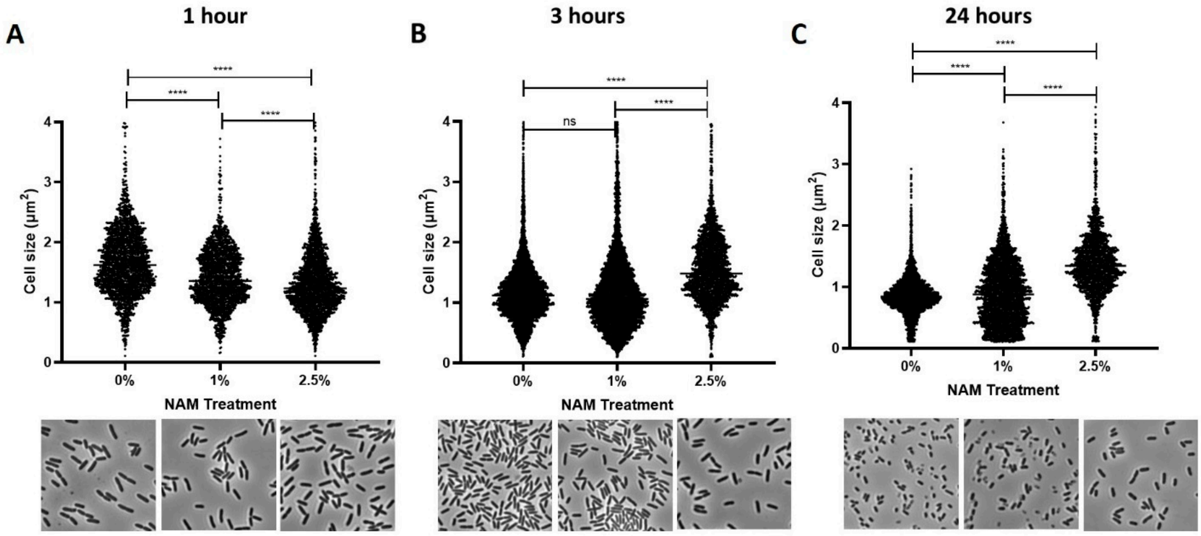

Figure S5. *P. aeruginosa* cell size analysis during incubation with 0, 1 and 2.5% niacinamide. Incubation periods were (**A**) 1h, (**B**) 3h and (**C**) 24h. Phase images of *P. aeruginosa* under niacinamide treatment at concentrations of 0, 1, 2.5% are located under the graphical measurements accordingly. (****=*p* value < 0.0001, ns= not significant).

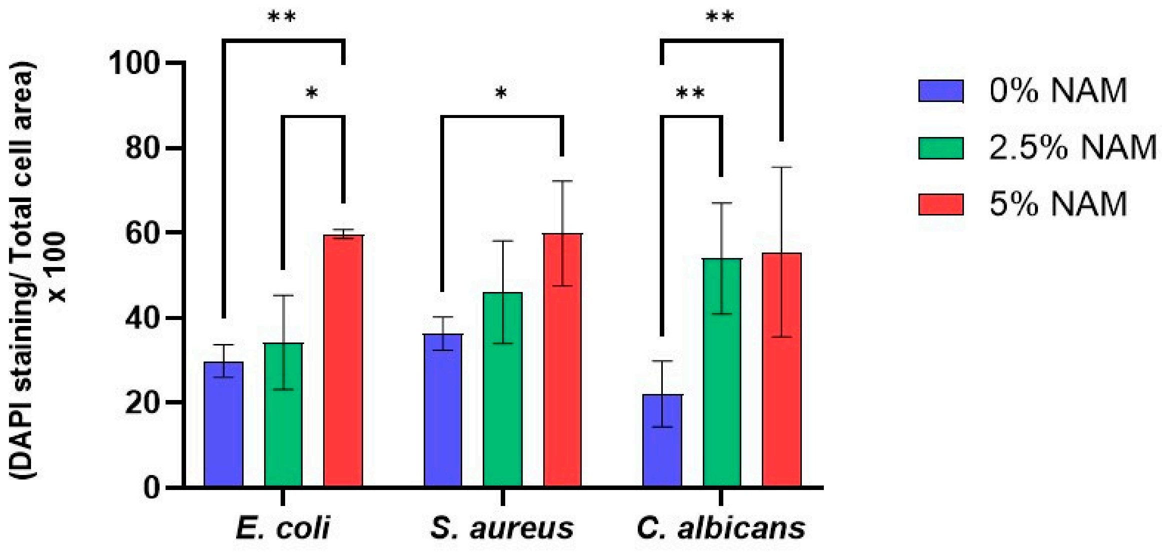

Figure S6. Quantitative analysis of DNA content by calculating DAPI staining area proportion out of the entire microbial cell area. *E. coli*, *S. aureus* and *C. albicans* DAPI stained fluorescence images, analyzed using ImageJ. (**= *p* value < 0.01, *= *p* value < 0.05).

microorganisms

MDPI

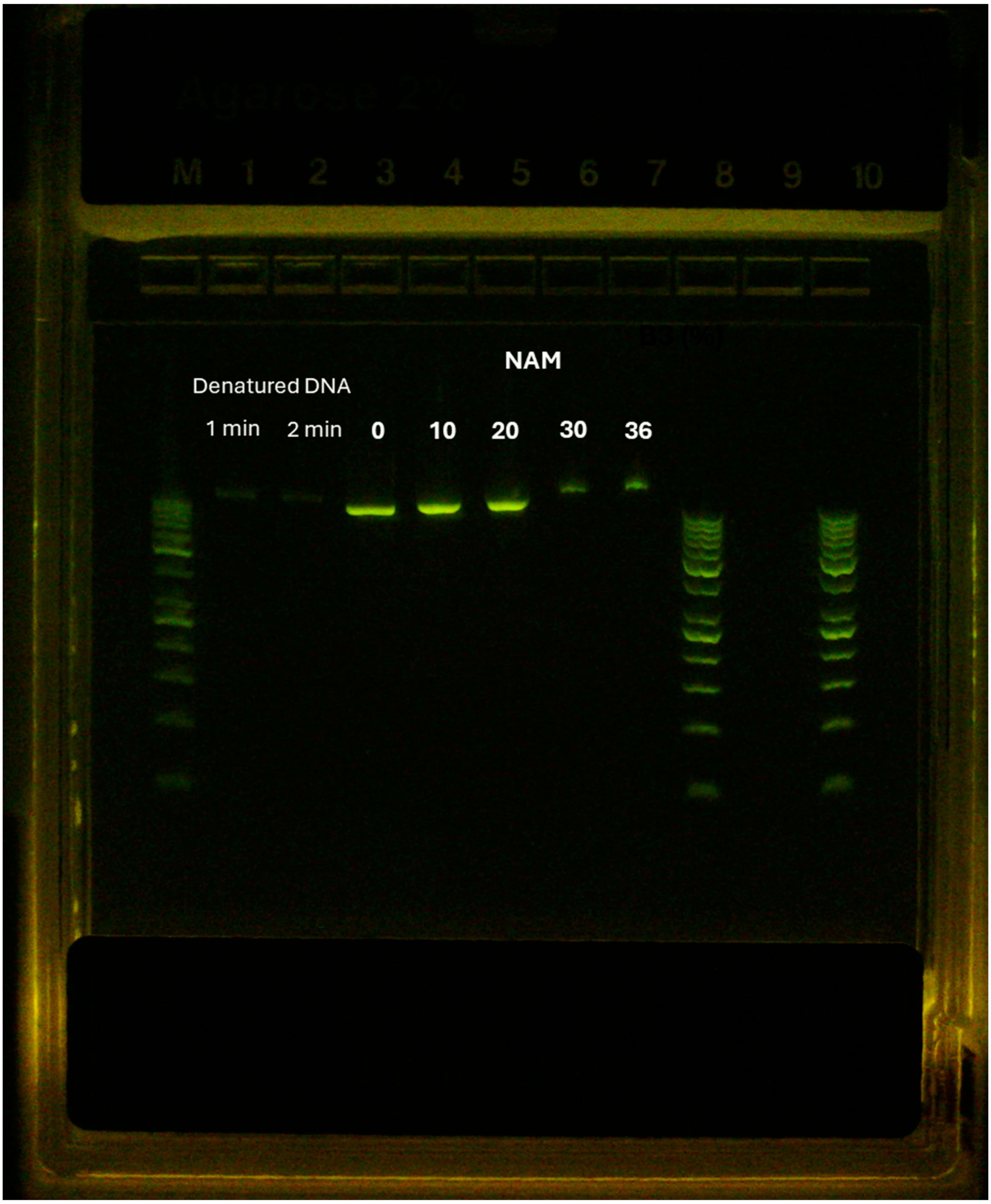

Figure S7: Original gel image. Niacinamide interaction with DNA amplicon by gel electrophoresis and qPCR. Agarose gel image of DNA fragment incubated with niacinamide at increasing concentrations.

microorganisms

MDPI

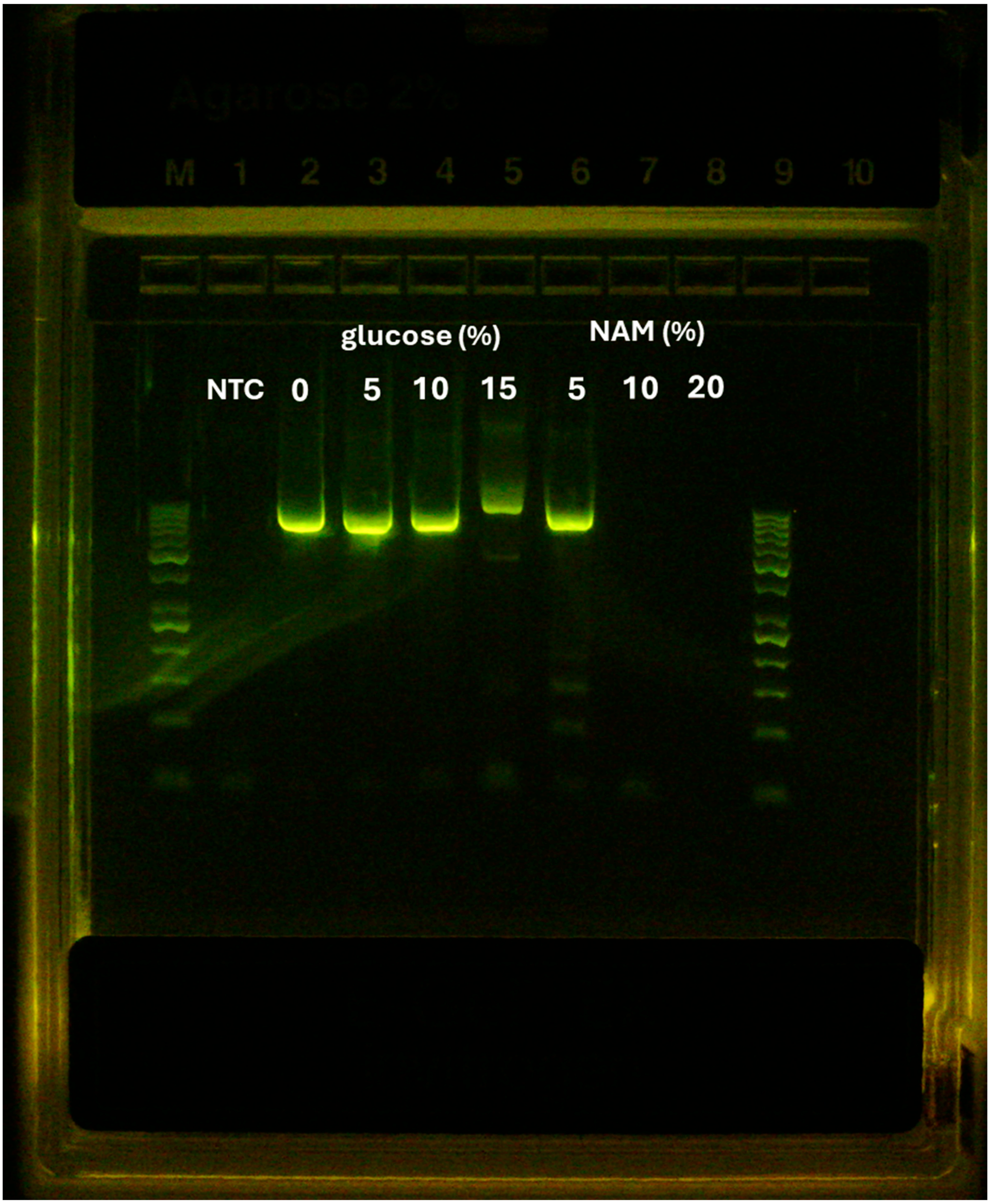

Figure S8: Original gel image. Clean PCR products of amplified DNA treated with increasing concentrations of niacinamide
